# MicroRNAs delivery into human cells grown on 3D-printed PLA scaffolds coated with a novel fluorescent PAMAM dendrimer for biomedical applications

**DOI:** 10.1038/s41598-018-32258-9

**Published:** 2018-09-17

**Authors:** Alessandro Paolini, Luca Leoni, Ilaria Giannicchi, Zeinab Abbaszadeh, Valentina D’Oria, Francesco Mura, Antonella Dalla Cort, Andrea Masotti

**Affiliations:** 1 Bambino Gesù Children’s Hospital-IRCCS, Research Laboratories, V.le di San Paolo 15, 00146 Rome, Italy; 2 Department of Chemistry, Sapienza University of Rome, P.le A. Moro 5, 00185 Rome, Italy; 3 Center for the Nanotechnology applied to the Engineering of La Sapienza (CNIS), Sapienza University of Rome, P.le A. Moro 5, 00185 Rome, Italy

## Abstract

Many advanced synthetic, natural, degradable or non-degradable materials have been employed to create scaffolds for cell culture for biomedical or tissue engineering applications. One of the most versatile material is poly-lactide (PLA), commonly used as 3D printing filament. Manufacturing of multifunctional scaffolds with improved cell growth proliferation and able to deliver oligonucleotides represents an innovative strategy for controlled and localized gene modulation that hold great promise and could increase the number of applications in biomedicine. Here we report for the first time the synthesis of a novel Rhodamine derivative of a poly-amidoamine dendrimer (G = 5) able to transfect cells and to be monitored by confocal microscopy that we also employed to coat a 3D-printed PLA scaffold. The coating do not modify the oligonucleotide binding ability, toxicity or transfection properties of the scaffold that is able to increase cell proliferation and deliver miRNA mimics (i.e., pre-mir-503) into human cells. Although further experiments are required to optimize the dendrimer/miRNA ratio and improve transfection efficiency, we demonstrated the effectiveness of this promising and innovative 3D-printed transfection system to transfer miRNAs into human cells for future biomedical applications.

## Introduction

In recent years, many studies have been focused on the preparation of three-dimensional (3D) biocompatible scaffolds for cell growth to be used as implantable systems for tissue engineering applications *in vivo*^1–3^. In particular, these engineered scaffolds are able to mimic an artificial extracellular matrix (ECM) and facilitate cell adhesion, proliferation, migration and guide tissue regeneration by host or transplanted cells^4^. For biomedicine applications, synthetic, natural, degradable or non-degradable materials have been employed to create scaffolds^5^. One of the most versatile and widely tested material is poly-lactide (PLA)^6^. PLA is a biodegradable and thermoplastic aliphatic polyester derived from corn starch that has been approved by the US Food and Drug Administration (FDA) for the direct contact also with biological fluids^7^. PLA has been previously assessed as copolymer for drug delivery systems^8^, wound healing^9^ and tissue engineering^10,11^.

Interestingly, the concept to employ the scaffolds themselves for gene delivery applications represents an innovative strategy for controlled gene delivery and localized transgene expression with great potential in many clinical fields. The first attempt to create scaffolds with specific delivery properties for tissue engineering applications appeared almost 20 years ago by Shea and collaborators that reported the controlled and sustained release of plasmid DNA from a biodegradable matrix^12^. Of course, to obtain an effective matrix for gene delivery and tissue regeneration application, many parameters such as the biomaterial choice, the cellular compatibility and the kind of gene vector (i.e., plasmid DNA or synthetic oligonucleotides) to promote cell growth should be accurately evaluated^13^. We reasoned that the important properties of a biocompatible scaffold to promote cell growth and deliver a transgene at the same time, would have afforded an innovative and peculiar material to employ in biomedical applications and tissue regeneration. Among the transgenes, microRNAs (miRNAs) are surely among the most attractive for their ability to regulate multiple genes at once. MiRNAs are a class of small endogenous non-coding RNAs that are able to regulate the gene expression post-transcriptionally and act as critical regulators of many biological processes within the cell, similarly to siRNA^14^. Unfortunately, such a multifunctional scaffold is not available so far.

Since many years, poly-amidoamine (PAMAM) dendrimers have been used as non-viral drug delivery systems for miRNAs^15^. PAMAM dendrimers have an inner alkyl-diamine core, commonly ethylenediamine, an amidoamine backbone and many terminal primary amine groups exploitable for bioconjugation with drugs or other targeting molecules^16^. At physiologic pH, PAMAM dendrimers are positively charged and have high affinity with nucleic acids, being good candidates for miRNA complexation and transfection into cells^17^. Therefore, these properties allow this polymers to be ideal carriers for anticancer drugs or intracellular gene delivery for cancer therapy applications^18–20^ and other biomedical applications^21^. However, the internalization of miRNAs/siRNAs or other nucleic acids by a fluorescent PAMAM derivative would greatly enhance the overall visualization of the transgene and the study of the overall internalization process.

**Figure 1:**
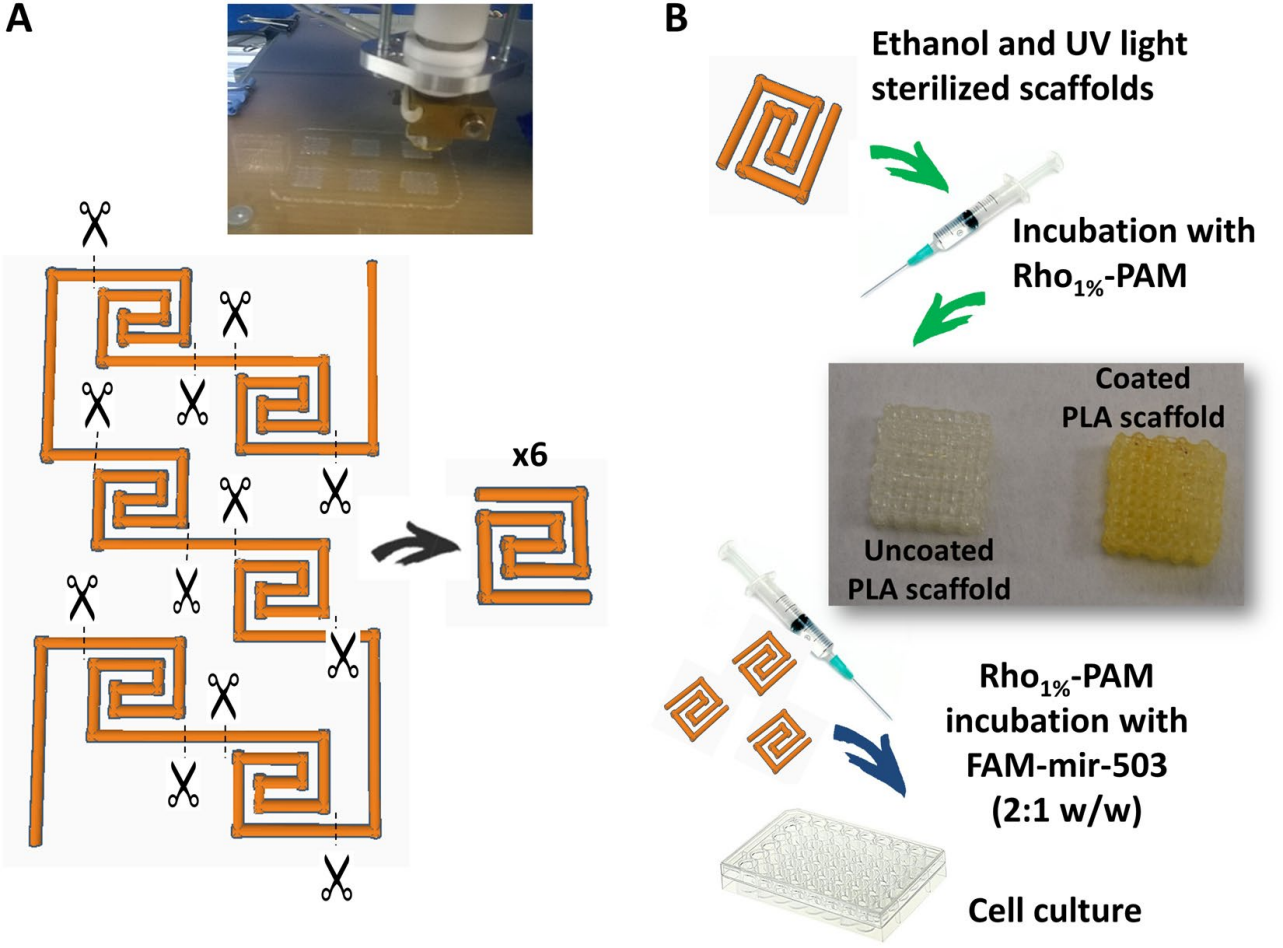
Schematic representation of the strategy adopted to obtain the 3D-printed scaffold. A larger structure has been printed and properly cut to obtain six scaffolds.

Therefore, the aim of the present work was to prepare for the first time a 3D-printed scaffold for cell growth covered by a novel fluorescent PAMAM dendrimer, obtained by conjugating it with a Rhodamine B label in order to evaluate the cell transfection ability of this advanced material (Fig. 1). The novelty of this work relies in the ability of cells to grow on this 3D structure allowing also to study the internalization of a fluorescent miRNA during their growth. Finally, we believe that, the innovative 3D-printed scaffold coated with the novel PAMAM fluorescent dendrimer that we have prepared will represent, in the next few years, a useful biomaterial for many biomedical applications and tissue engineering applications.

## Results

### PAMAM functionalization with Rhodamine B

**Figure 2:**
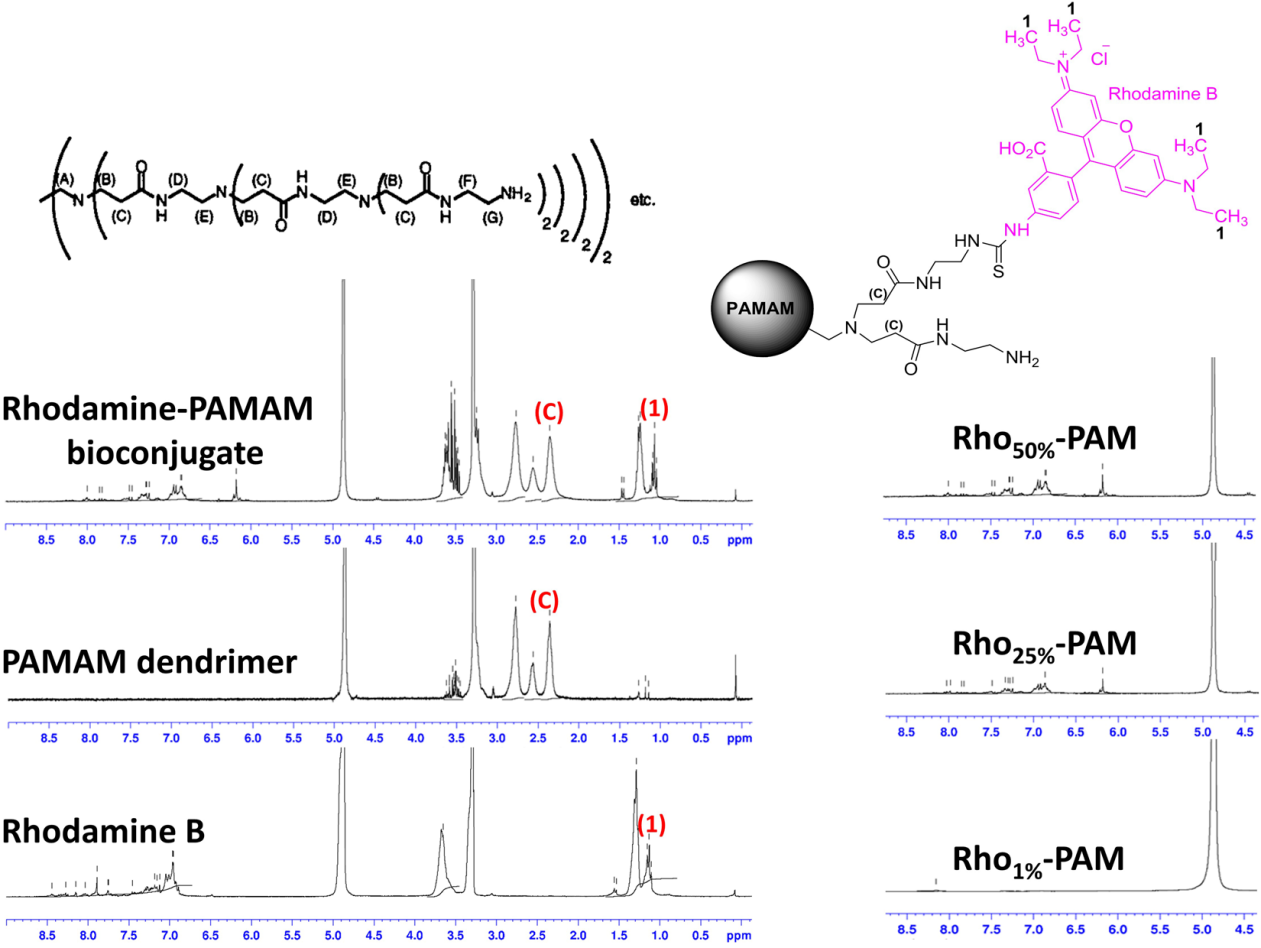
^1^H NMR spectra in CD_3_OD of Rhodamine B isothiocyanate (bottom left), PAMAM dendrimer (G = 5) (middle left), Rhodamine-PAMAM (Rho-PAM) bioconjugate (upper left). On the right, a magnification of the aromatic protons of the derivatives: Rho_1%_-PAM (bottom right), Rho_25%_-PAM (middle right), Rho_50%_-PAM (upper right). All proton resonances were assigned according to the scheme depicted in the upper part of the picture.

To calculate the percentage of dye functionalization of PAMAM dendrimer, we acquired ^1^H NMR spectra and we integrated separately the peaks belonging to rhodamine B and to dendrimer, according to the scheme reported in Fig. 2. The peak (C) in Fig. 2 represents the α-protons adjacent to carbonyl groups of PAMAM dendrimer, (for a total number of 504 protons). Integral (1) integrates the twelve methyl protons of rhodamine B moiety. Therefore, by calculating the ratio of these two integrals we were able to obtain the percentage of substitution of the three Rho-PAM compounds. In particular, for the three nominal derivatizations (i.e. 1%, 25% and 50%) we obtained the following three percentages of substitution: 1.2%, 23.8% and 43.7%. For clarity, in the manuscript we referred these compounds as Rho_1%_-PAM, Rho_25%_-PAM and Rho_50%_-PAM, respectively.

### 3D printing afforded biocompatible PLA scaffolds

Classic 3D printing by extrusion of a plastic filament (i.e. PLA, ABS, PET, PETG, etc.), referred as fused filament fabrication or FFF, is commonly seen as a mean to produce macroscopic objects for daily use. However, some of these filaments can be also exploited for their biocompatibility. The extrusion of PLA filaments represents a simple and affordable technique to produce good scaffolds for biomedical applications. One of the most important aspect to consider during the fabrication of these objects is the presence of bubbles or cracks formed after the filament extrusion, due to continuous stop-and-start printing of the scaffolds. These imperfections create structural defects that may prevent the downstream application of these printed models. For these reasons, we decided to join many spiral scaffolds in a single bigger model in order to reduce imperfections during the printing step. The overall model was cut at fixed points, thus obtaining six homogeneous spiral scaffolds that were employed for further experiments (Fig. 1A). The main properties of the PLA scaffolds were reported in Table S1. The characteristics of the scaffolds were used to calculate the amount of PAMAM needed for the scaffold functionalization and to evaluate the number of cells to use for seeding.

### Coating of PLA scaffolds with Rho_1%_-PAM

**Figure 3:**
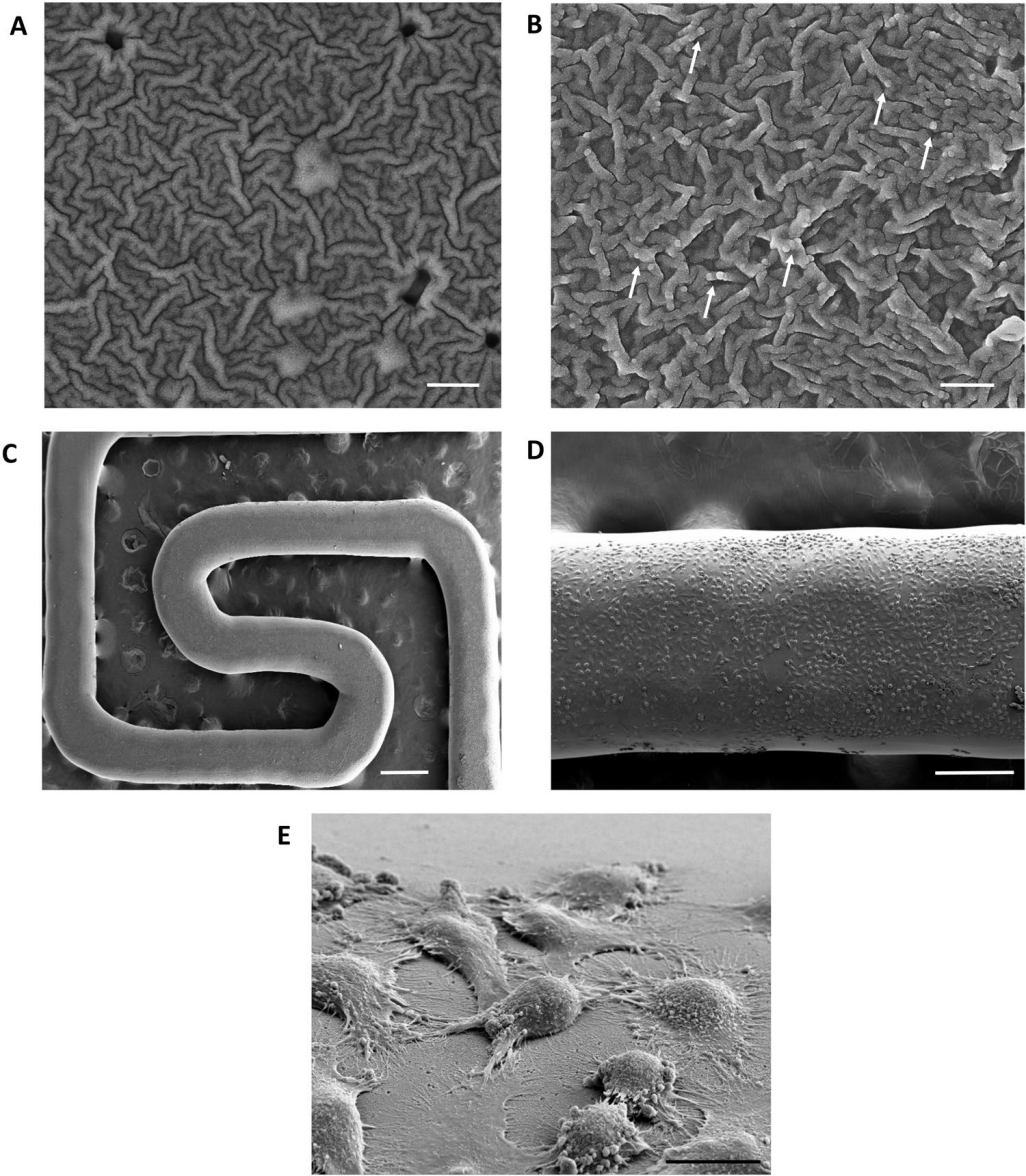
SEM images of PLA scaffold surface alone (**A**) and after incubation with Rho_1%_-PAM solution (**B**). The white arrows show some dendrimer aggregates. Magnification 50.00 Kx, scale bar 400 nm. (**C**–**E**) HeLa grown on PLA scaffold coated with Rho_1%_-PAM at different magnification: (**C**) 60X, scale bar = 500 µm; (**D**) 250X, scale bar = 200 µm and (**E**) 5.54 Kx, scale bar = 10 µm.

PLA scaffolds were coated with Rho_1%_-PAM resulting in a final macroscopic structure that did not differ substantially from the uncoated scaffold (Fig. 3A,B and Supplementary Fig. S1). We decided to use Rho_1%_-PAM for all downstream analyses, since higher percentages of substitution yielded very fluorescent compounds that gave an aberrantly high signal in confocal microscopy analyses, preventing a clear examination. Among the strategies used to monitor qualitatively the adhesion of Rho_1%_-PAM to PLA scaffolds, we decided to use a simple chemical reaction (i.e., iodine vapours), one of the methods for the visualization of amine groups. After incubating the scaffolds in the presence of iodine vapours, the coated scaffolds reacted with iodine to give a yellow colour (Fig. 1B). This technique was very sensitive since the yellow colour developed easily also when we coated the scaffold with the Rho_1%_-PAM derivative. To quantify the amount of Rho_1%_-PAM bound to the scaffold surface, we performed a preliminary thermogravimetric analysis (TGA) on PAMAM coated PLA scaffolds without obtaining exploitable results owing to the superimposition of the thermogravimetric curves of PLA and PAMAM during heating (data not shown). Therefore, to overcome these limitations, we determined semi-quantitatively the amount of Rho_1%_-PAM bound on the PLA surface by spectrophotometric analysis. A calibration curve allowed us to determine the amount of free Rho_1%_-PAM in solution after incubation of the functionalized polymer with the scaffold (data not shown) and calculate by difference the amount of Rho_1%_-PAM bound to it. Therefore, considering a mean weight of 6.0 ± 0.4 mg for each scaffold, the amount of Rho_1%_-PAM on its surface was ~30 µg (less than 1% w/w). This amount allowed us to calculate the optimal weight ratio of Rho-PAM/FAM-mir-503 to use in transfection experiments.

### Ultrastructural SEM and EDX analyses

In order to visualize in details the potential structural surface modifications of PLA scaffolds after the functionalization, we performed SEM and EDX analyses. The PLA scaffold surface appeared porous with rare small fractures, affording a ‘wrinkly’ surface (Fig. 3A). The coating of PLA scaffolds with PAMAM is highlighted by SEM by the presence of few small polymer aggregates that are not visible in the control sample (i.e., scaffolds maintained at the same conditions as the coated scaffolds, without PAMAM) (Fig. 3B). The EDX analysis also confirmed the presence of PAMAM on the scaffold surface owing to the presence of nitrogen in the elemental mapping (%N = 2.11 ± 0.23), which was slightly higher compared to PLA scaffold alone (%N = 0.62 ± 0.23) although the values were at the limit of the instrumental sensitivity/resolution (Figure S2). SEM analysis allowed also to observe many healthy cells attached to the coated PLA scaffold (Fig. 3C,D) that at higher magnifications showed several extroflections/pseudopodia (Fig. 3E). This suggested a strong interaction between cells and the surface of the scaffold. The functionalization of PLA scaffold with Rho_1%_-PAM did not significantly change the cell morphology (Fig. 3E). We believe that the adhesive properties of PAMAM dendrimer coupled to a rough scaffold surface could increase the cell/scaffold reciprocal interactions improving the use of PLA-scaffolds as a supporting material for cell adhesion^22^.

### Cell proliferation on PLA scaffolds coated with Rho_1%_-PAM

**Figure 4:**
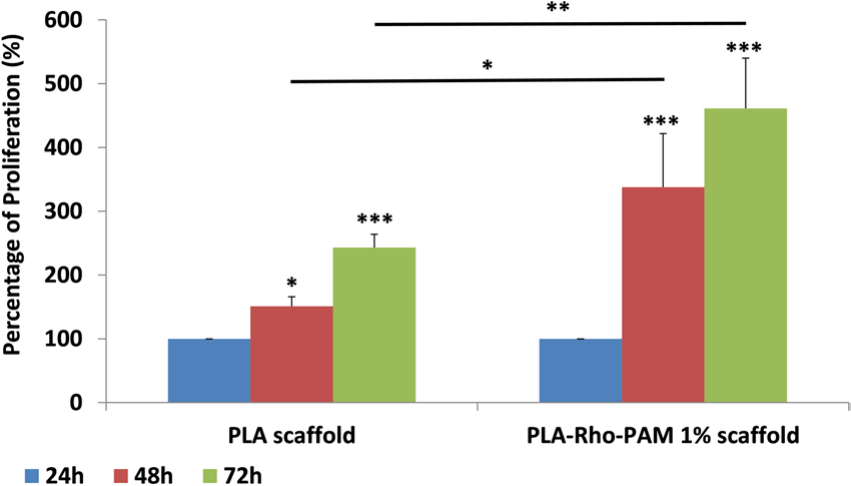
Proliferation assay for HeLa cells grown on PLA scaffold (on the left) and on PLA scaffold coated with Rho_1%_-PAM. Values are expressed as Mean ± SE (n = 3). (For *p < 0.05, for **p < 0.01, for ***p < 0.005).

To quantify the proliferation of HeLa cells on PLA scaffold coated with Rho_1%_-PAM we employed MTT assay to measure the intensity of formazan salts produced by proliferating cells after 24, 48 and 72 hours of incubation upon the coated scaffolds. The scaffolds resulted to be a good substrate to seed the cells and not cytotoxic at all. In fact, we observed a statistically significant increase of cell proliferation as a function of time, outlining a stable interaction between the cells and the scaffold (Fig. 4). Interestingly, by normalizing the percentage of cells proliferating on PLA scaffolds at 24 hours (100% of proliferation), after 48 and 72 hours we obtained very different results with coated or uncoated PLA scaffolds. In particular, after 48 and 72 hours cells adhering on uncoated PLA scaffold displayed a proliferation increase of 150 and 250%, respectively, whereas on Rho_1%_-PAM -coated scaffolds, the percentage is significantly higher (320 and 450%, respectively). These data greatly emphasize the importance to employ PAMAM dendrimers to increase the amount of cells attaching and proliferating on the PLA scaffold structures^23,24^.

### Rho-PAM dendrimer is able to bind miRNAs

**Figure 5:**
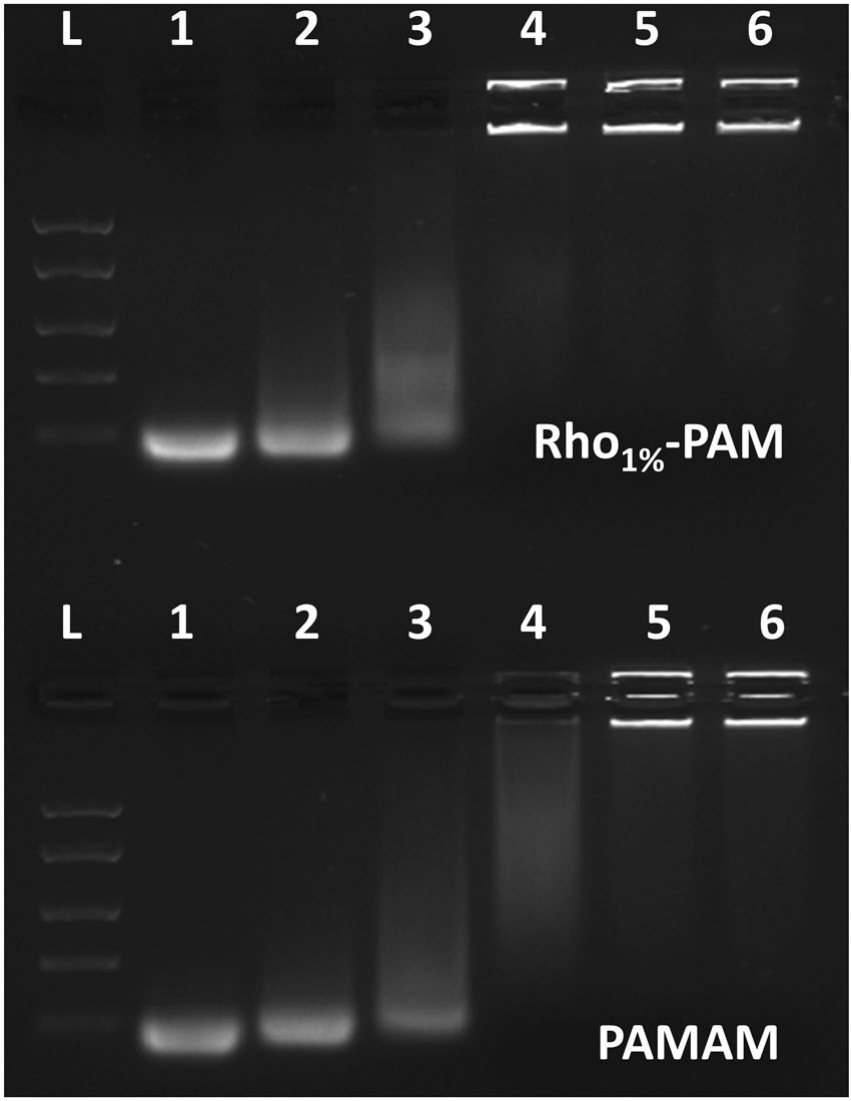
Agarose gel (1%) of Rho_1%_-PAM/miRNA (0.5:1, 1:1, 2:1, 4:1 and 8:1 w/w ratios) complexes (lane 2–6, respectively) (up) and PAM/miRNA (0.5:1, 1:1, 2:1, 4:1 and 8:1 w/w ratios) complexes (lane 2–6, respectively) (bottom). Ladder (lane L) and miRNA alone (lane 1) were run as controls.

A recent study from our group assessed the ability of PAMAM to bind effectively a ~70 nt oligonucleotide (oligo) mimicking a pre-miRNA (hsa-mir-503) for the delivery of non-coding RNAs into cells^25^. To assess the ability of PLA scaffold coated with Rho_1%_-PAM to bind small nucleic acids (i.e., oligonucleotides, miRNA mimic, etc.), we performed agarose gel retardation assays on pure polymers (coated and uncoated) (Fig. 5). Similarly, not only PAMAM alone but also the Rho_1%_-PAM dendrimer is able to complex miRNAs giving large aggregates unable to migrate into the gel. PAMAM/miRNA ratio of 0.5:1, 1:1 and 2:1 (w/w) led only to a partial complexation, whereas the 4:1 and 8:1 w/w ratios produced large aggregates quantitatively. Conversely, the Rho_1%_-PAM/miRNA 2:1 w/w ratio differed from the PAMAM/miRNA complex at the same ratio, leading to a quantitative complex formation. This suggest that the functionalization with Rhodamine moiety, although at a very low percentage of substitution, is able to modify the physicochemical properties of the polymer itself, ultimately leading to a stronger interaction with miRNAs. In conclusion, we can suppose that also the Rho-PAM-coated PLA scaffold should retain the ability to bind miRNAs.

### Rho-PAM/miRNA complex is able to translocate into HeLa cells

**Figure 6:**
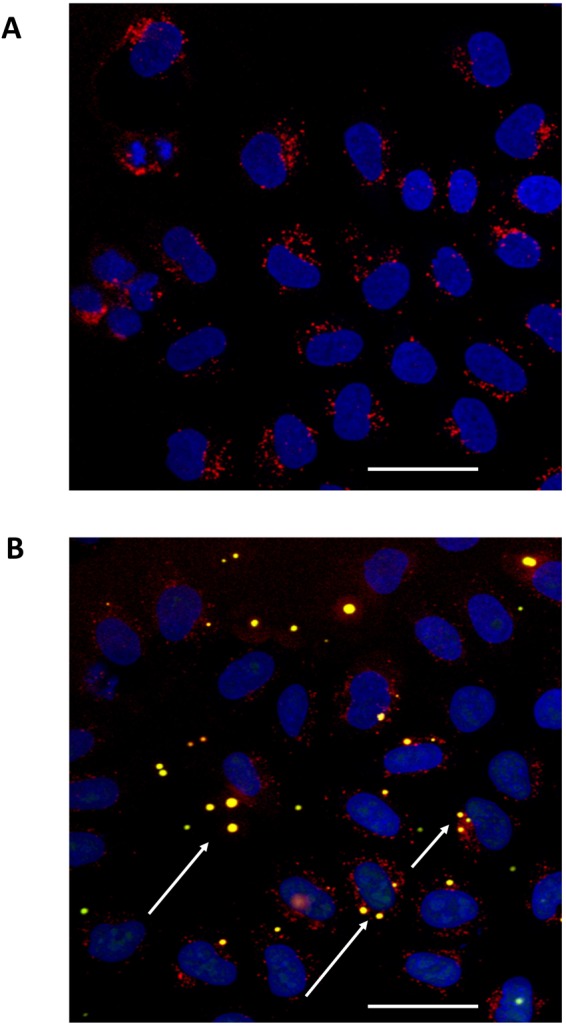
Fluorescence images of HeLa cells after treatments with Rho_1%_-PAM solution alone (**A**) and complexed with FAM-mir-503 (4:1 w/w ratio) (**B**). The red fluorescence indicates the dendrimer while the green fluorescence the mir-503 mimic. The yellow spots (white arrows) represent the Rho_1%_-PAM/miRNA complex within the cell. Magnification 40×, scale bar 50 µm.

In order to assess the effectiveness of Rho_1%_-PAM to deliver miRNAs mimics into mammalian cells, we treated HeLa cells with Rho-PAM/miRNA complexes at different transfection conditions. Rho_1%_-PAM was analysed both in solution as a pure polymer and bound to PLA scaffolds. The fluorescent miRNA mimic (FAM-mir-503) was used at different weight ratios (Rho_1%_-PAM:miRNA weight ratios of 2:1 and 4:1). The miRNA delivery was evaluated 24 h post-transfection by confocal fluorescence microscopy. Interestingly, Rho_1%_-PAM alone was able to enter into cells as represented by red dots in Fig. 6A. Similarly, also the miRNA complex with Rho_1%_-PAM displayed an optimal transfection efficiency and many vesicles were observed within the cell cytoplasm (Fig. 6B). Conversely, FAM-mir-503 alone was not able to enter into cells (Fig. S3). Therefore, the functionalization of PAMAM dendrimer with Rhodamine B dye allowed us to obtain a multifunctional system able not only to be monitored during the internalization into cells, but also to follow the delivery of miRNAs. This functionalization significantly improved the commercial system already assessed in our previous works^25,26^ and proved that the functionalization did not alter significantly its transfection and toxicity properties.

### 3D-printed PLA scaffold coated with Rho_1%_-PAM is able to transfect miRNAs into cells

**Figure 7:**
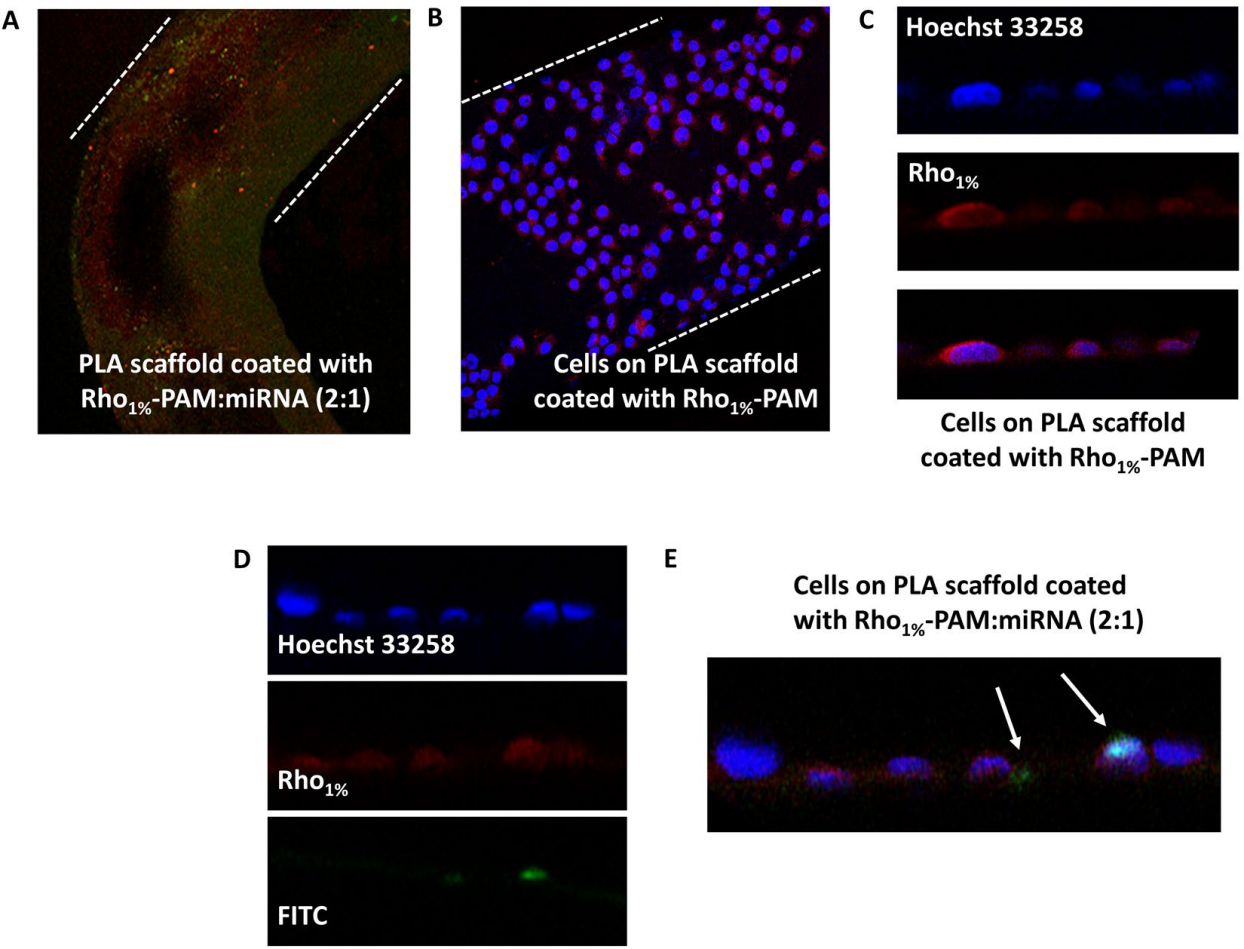
Confocal microscopy images of: (**A**) 3D-printed scaffold coated with Rho_1%_-PAM complexed with FAM-miRNA (magnification 10X); (**B**) HeLa cells grown on PLA scaffold coated with Rho_1%_-PAM (magnification 20X); (**C**) Magnification of HeLa cells (nuclei in blue) grown on PLA scaffold coated with Rho_1%_-PAM (red) (magnification 40X); Magnification (**D**) and overlay images (**E**) of HeLa cells (nuclei in blue) grown on PLA scaffold coated with Rho_1%_-PAM (red) complexed with FAM-miRNA (green) (magnification 40X).

After the 3D printing of PLA scaffolds, we decided to incubate them with the novel fluorescently-labelled PAMAM dendrimer (Rho_1%_-PAM) and assess its effectiveness to bind cells and transfect miRNAs into them. By confocal microscopy we were able to observe that the PLA scaffold was coated with the Rho_1%_-PAM compound (Fig. 7A) and that cells are able to grow on it (Fig. 7B–D). By growing cells onto the Rho_1%_-PAM-coated PLA scaffold incubated with FAM-mir-503, we observed the translocation of the fluorescent miRNA into cells (Fig. 7E). This finding support the use of dendrimer-coated PLA scaffolds as advanced materials for miRNA delivery into cells for biomedical and tissue engineering applications.

## Discussion

In the biomedical field, the use of 3D printing to realize objects for cell growth/proliferation has been generally performed through the use of biocompatible sacrificial gels (i.e., bio-inks) embedded with cells, that are surrounded by a permanent solid material which imparts the final form to the object. In some cases, these bio-inks can be removed to create hollow structures. In this work, we coupled two different technologies: the fused filament fabrication (FFF), more generally referred as 3D printing, and drug delivery with innovative non-viral vectors. In particular, we exploited the potential of 3D printing to design and realize objects that were further customized and functionalized with poly-amidoamine dendrimers to create multifunctional objects. The advantage of our approach was to create a customized structure (in our case a classic hierarchical scaffold) made by a biocompatible high-quality PLA. The shape of our PLA scaffold consisted of a hierarchical scaffold traditionally employed to create ‘self-assembling’ building blocks, or bio-assembly, that has been also defined a ‘bottom-up’ approach in the literature^27,28^. Following this approach, even different cell types can be embedded into one or more scaffold layers that after a layer-by-layer (LBL) assembly can form a homogeneous cell colony^29^. The addition of delivery abilities to the scaffold, such as in our system, could further facilitate cell colonization and proliferation as it would allow to control the microenvironment inside the scaffold in a better  way, as already observed in a previous LBL approach^30^.

The PLA scaffold was coated with a poly-amidoamine dendrimer (i.e., PAMAM G = 5) that we previously reported to have a high ability to transfect endothelial cells with microRNAs^26^. Moreover, this dendrimer seems to have also adhesive properties thus facilitating the adhesion and proliferation of cells adhering to coated surfaces.

In this work, we report for the first time the synthesis and the characterization of a novel PAMAM derivative, a Rhodamine-labelled PAMAM conjugate, which allowed a real-time visualization of the transfection process. The internalization of Rho-PAM, assessed by the intense red fluorescence inside the cells (Fig. 6A), validate the use of this PAMAM derivative as a novel non-toxic delivery vector^31,32^.

Thus, by preparing a 3D-printed scaffold coated with a fluorescently-labelled dendrimer, we were able to obtain a biocompatible multifunctional scaffold for cell proliferation and transfection with miRNAs. This approach revealed successful, especially for long-term (i.e., 48 and 72 h) growing of cells and suggests the use of these innovative scaffolds also in the field of tissue engineering. Moreover, poly-amidoamine dendrimers can be easily functionalized with other molecules (i.e., other dyes or other functional molecules), paving the way to the realization of multifunctional materials for a controlled delivery of drugs, grow factors or other therapeutics.

**Figure 8:**
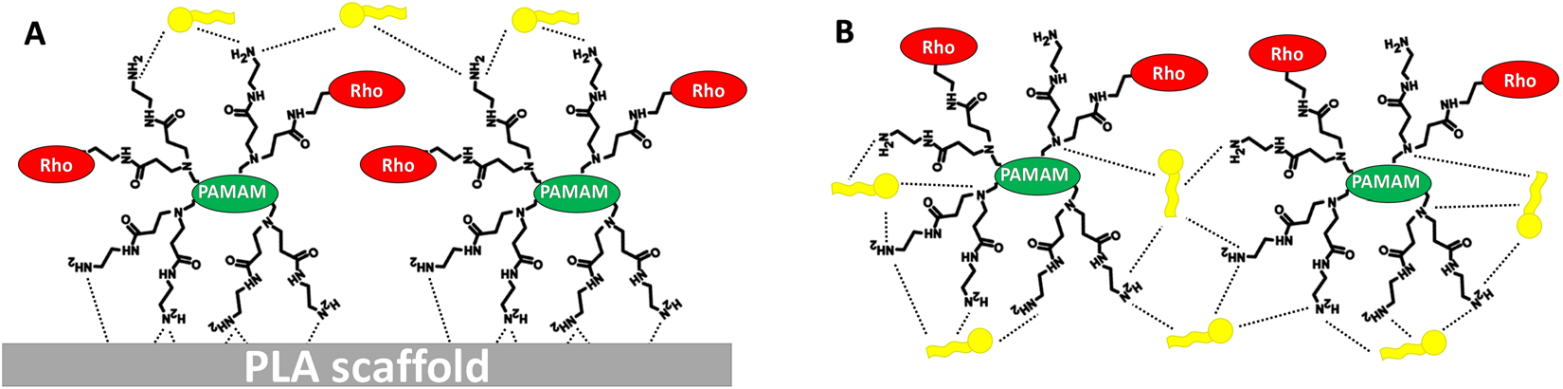
Schematic representation of the transfection process by dendrimers layered on a rigid surface (**A**). The interaction of Rho-PAM terminal amino groups with the PLA scaffold may weaken the interaction with miRNAs (yellow), being many groups involved in the adhesion with the scaffold. On the contrary, the poly-amidoamine dendrimer in solution (**B**) has many more ‘degrees of freedom’ and can more easily interact with miRNAs and form more stable complexes (or dendriplexes).

In our system, the delivery of mir-503 mediated by the three-dimensional scaffold was not observed in all cells. We hypothesize that the transfection process by dendrimers layered on a rigid surface may have different binding and release properties. In fact, being the PLA scaffold a rigid micrometric structure, Rho-PAM covered just only the outermost part of it (Fig. 8). As depicted in Fig. 8, the interaction of Rho-PAM terminal amino groups with the PLA scaffold may weaken the interaction with the miRNA, being many groups involved in the adhesion with the scaffold (i.e., by electrostatic and/or Van del Waals interactions) or simply ‘covered’ by the overlying polymer coating. On the contrary, the poly-amidoamine dendrimer in solution has many more ‘degrees of freedom’ and can more easily interact with miRNAs and form more stable complexes (or dendriplexes). As a consequence, also the weight ratio between the polymer and the miRNA should be optimized in order to obtain an efficient ‘scaffold-supported’ delivery vector. Although we have not optimized this ratio, nevertheless we demonstrated the proof of concept of this promising and innovative 3D-printed system.

## Conclusions

In this work, we synthesized for the first time a novel fluorescent derivative of PAMAM dendrimer, a functional compound able to track *in vitro* the delivery of nucleic acids. In fact, this dendrimer is able to complex miRNAs similarly to the pristine compound. This novel compound has been also employed to cover a 3D-printed PLA scaffold to afford an advanced material able to facilitate cell growth and proliferation on its surface. This novel material is biocompatible, easily and quickly produced by standard techniques, and customizable by linking other therapeutic compounds on its surface, leading to multifunctional healthcare materials. The advanced material produced in this work can be used not only in the biomedical field but also potentially employed in tissue regeneration applications.

## Methods

Rhodamine B isothiocyanate, PAMAM dendrimer (G5), sodium bicarbonate, CD_3_OD, FAM-labelled oligonucleotide mimicking the precursor form of miRNA-503 (FAM-mir-503, batch no. HA08019806), thiazolyl blue tetrazolium bromide (MTT, cat no. M5655), Hoechst 33258 (Sigma-Aldrich, cat. No. 861405) were purchased by Sigma-Aldrich and used as received. 10 K Microcon Centrifugal Filter Devices were purchased by Millipore (Billerica Massachusetts, USA) and PVDF syringe filter by Whatman (Maidstone, UK). Poly-lactide (PLA) filament was from Shenzhen Esun Industrial Co., Ltd, China. The 3D printer 3DRag was from Futura Group srl, Italy.

### 3D printing of PLA scaffolds

A transparent and recyclable PLA filament was used to obtain an easy-to-print scaffold for cell culture, designed by using the Thinkercad application, a straightforward browser-based 3D design and modelling tool (www.tinkercad.com, ©2016 Autodesk, Inc.), and extruded using an assembled 3D printer (3D Rag) (Fig. 1A). To print the PLA scaffolds, the extrusion temperature was set to 193 °C, whereas the bed temperature at 55 °C. The design allowed to have multiple spiral-shaped scaffolds having only one layer (500 µm), easily printable and manageable for downstream experiments (i.e., confocal microscopy). Moreover, the spiral shape of the structure (Fig. 1A) allowed to obtain multiple scaffolds rapidly (printing time = 80 sec) on a limited printing area and maximize the surface area of each scaffold for cell growth.

### Synthesis of Rhodamine-labelled PAMAM dendrimer (Rho-PAM)

PAMAM dendrimer G5 has 128 reactive terminal amino groups that can be functionalized with amino-reactive probes, such as isothiocyanates. To obtain dye-labelled PAMAM compounds at different percentage of substitution, PAMAM (20 mg) was dissolved in 1 ml of sodium bicarbonate aqueous solution. An amount of 23.5, 587.5 and 1175 µl of a rhodamine B isothiocyanate solution in methanol (20 mg/ml) was added to obtain a calculated rhodamine-labelled PAMAM (Rho-PAM) substitution of 1%, 25% and 50%, respectively. The three derivatives were purified by filtering the crude compounds (yield 98.5%) through Microcon spin filters. Compounds were re-suspended in nuclease free water and sterilized by 0.45 µm PVDF filter. The characterization of Rho-PAM compounds was carried out by ^1^H-NMR spectroscopy in CD_3_OD and recorded on a Bruker AC300P spectrometer.

### Qualitative and quantitative evaluation of Rho-PAM amount on PLA scaffolds

To evaluate qualitatively the amount of Rho-PAM bound to PLA scaffolds, the iodine vapours technique was performed. Briefly, PLA scaffolds coated with Rho_1%_-PAM were washed in distilled water and dried in a glass chamber. Few iodine crystals (Sigma Aldrich, Milan, Italy) were added inside the container and the chamber was heated until iodine vapours were observed. Uncoated PLA scaffolds were treated similarly for comparison purposes (Fig. 1B). To evaluate quantitatively the amount of Rho_1%_-PAM bound to PLA scaffolds, we determined the free Rho-PAM (after incubation) by spectrophotometric determination (580 nm) and we calculated the amount of bound Rho_1%_-PAM by difference (Fig. S4).

### Surface functionalization of PLA scaffolds with Rho-PAM dendrimer

The 3D printed PLA scaffolds were carefully weighted prior to incubation with Rho-PAM dendrimer. We decided to start the incubation with Rho_1%_-PAM at a scaffold:dendrimer weight ratio of 100:1. Similar results were also obtained by using Rho-PAM at higher functionalization percentages. Approximately 6 mg of PLA scaffolds were incubated with Rho_1%_-PAM solution (60 µg) in PBS (150 µl) inside a 2.5 ml syringe in order to avoid the formation of air bubbles, obtain a homogeneous functionalization and facilitate the following washing steps (Fig. 1B). After 3 days of incubation, the scaffolds were washed with sterile PBS and further incubated for 3 hours with FAM-mir-503 (~4.5 µl, 2.27 µg/µl) at a Rho_1%_-PAM:miRNA ratio of 2:1 w/w compared to PLA in order to proceed with the *in vitro* experiments (Fig. 1B).

### ^1^H NMR characterization of Rho-PAM derivatives

Rho-PAM derivatives (functionalized at 1%, 25% and 50%) were dissolved in CD_3_OD and ^1^H NMR spectra were recorded on a Bruker AC300P spectrometer. The spectra of pure PAMAM and Rhodamine B isothiocyanate dissolved in the same solvent were also recorded for comparison purposes (Fig. 2). Peaks were assigned according to a method previously reported and depicted in Fig. 2 ^33^.

#### Gel retardation assays

Agarose gel retardation assays were carried out following standard procedures^34^. PAM, Rho_1%_-PAM and FAM-mir-503 (50 nM) were mixed at dendrimer/miRNA ratios of 0.5:1, 1:1, 2:1, 4:1 and 8:1 (w/w) and incubated at room temperature for 15 min. The complexes were loaded into 1% agarose gel (in TAE buffer) and run for 30 min at 90 V. Hoechst 33258 (10 mg/mL) was added to the agarose gel as intercalating dye and gels were visualized by UV light (Fig. 5).

### Adhesion of HeLa cells on PLA scaffolds

The PLA scaffold was layered onto a thin film of 1% agarose prepared in a 48-well plate. Agarose was employed to avoid undesirable adhesion of cells onto the bottom of the culture plate^29^ and to prevent the floating of the PLA scaffold itself in the culture medium. For each PLA scaffold, 4 × 10^5^ cells dispersed into 75 µl of serum free medium were placed onto the scaffold and 600 µl of complete medium were added after 3 h.

### Scanning Electron Microscopy of PLA scaffolds

Cells grown on PLA scaffolds with or without Rho_1%_-PAM coating were fixed with 2.5% glutaraldehyde in 0.15 M cacodylate buffer (pH 7.2) for 2 h at room temperature. After dehydration with an alcoholic gradient, samples were coated with chromium (30 nm) using a Quorum 150 T sputter. Field emission scanning electron microscopy (FESEM) analysis was performed using a Zeiss Auriga microscope, operating at a low acceleration voltage and current in order to avoid beam damages of the cells (Fig. 3).

### Proliferation and transfection of HeLa cells grown on PLA scaffolds

To quantify the ability of HeLa cells to grow on PLA scaffolds with or without Rho_1%_-PAM coating, a MTT proliferation assay (Sigma Aldrich, Milan, Italy) was performed. After 24, 48 and 72 h from cell seeding, the amount of cells on both type of PLA scaffolds (coated and uncoated) was measured (Fig. 5). PLA scaffolds were washed with PBS and incubated with MTT solution (5 mg/ml). After 3 h, the medium was removed and the obtained formazan crystals were dissolved in 200 µl of pure dimethyl sulfoxide (DMSO). The absorbance (570 nm) of formazan crystals was measured by an ELISA plate reader (Benchmark Plus, BIO-RAD) and background (630 nm) subtracted.

### Transfection of HeLa cells grown on PLA scaffolds

To monitor the ability of PLA scaffolds coated with Rho_1%_-PAM to transfect cells compared to the free Rho_1%_-PAM compound, two different protocols were performed. For transfection with free Rho_1%_-PAM, cells grown on a glass cover slip were treated with 0.8 µg of Rho_1%_-PAM and 0.2 µg FAM-mir-503, previously incubated for 15 min, in serum free medium. After 4 h, the solution was replaced with 500 µl of complete medium and incubated for 24 h. To monitor transfection of PLA scaffolds coated with Rho_1%_-PAM, cells were seeded directly on the scaffold in serum free medium and incubated for 4 h. After 4 h, the solution was replaced with 600 µl of complete medium and incubated for 24 h. After 24 h, cells were washed one time with cold phosphate buffered saline (PBS) and fixed with 4% parafomaldehyde solution for 15 min at room temperature. After a washing step with PBS, nuclei were stained by incubating cells with a Hoechst 33258/PBS solution (1:2500) for 3 min. Fixed cells were mounted with a glycerol/PBS solution (3:1) and kept covered to prevent dye photo-bleaching before fluorescence image acquisition. Slides were acquired with a Olympus Fluoview FV1000 confocal microscope (Olympus, Italy) equipped with FV10-ASW version 4.1a software, Multi Ar (458–488 and 512 nm), 2X he/Ne (543 and 633 nm) and 405-nm diode laser, using 40x (N.A 0.90) objective and 60x (N.A 1.42) oil objective. Optical single sections were acquired with a sequential scanning mode format of 1024 × 1024 pixels and sampling speed of 20µs/pixel. Cells grown on PLA scaffolds were acquired with a Leica TCS-SP8X laser-scanning confocal microscope (Leica Microsystems, Mannheim, Germany) equipped with tunable white light laser (WLL) source, 405 nm diode laser, 3 Internal Spectral Detector Channels (PMT) and 2 Internal Spectral Detector Channels (HyD) GaAsP, 10X and 20X (N.A 0.4 and 0.7) magnifications. Z-reconstructions of serial single optical sections were performed with a sequential scanning mode of 1024 × 1024 pixels, scan speed of 400 Hz, and z-step size of 2.5 μm.

### Statistical analysis

Statistical comparison between various groups was performed by Student’s t-test or one-way analysis of variance (ANOVA) with either least significant difference (LSD) post hoc tests, using the SPSS software (12.0.2). Comparisons were made between means from several experiments. Differences were considered significant when *p* values were <0.05. Statistical significance is indicated with for *p < 0.05, for **p < 0.01, for ***p < 0.005.

## Electronic supplementary material

Supporting Information
