# Supplementary material for: MicroRNAs delivery into human cells grown on 3D-printed PLA scaffolds coated with a novel fluorescent PAMAM dendrimer for biomedical applications
Source: Sci Rep. 2018 Sep 17;8:13888. doi: 10.1038/s41598-018-32258-9 (PMC6141561; doi:10.1038/s41598-018-32258-9)

**Supporting Information**

**MicroRNAs delivery into human cells grown on promising 3D-printed PLA scaffolds coated with a novel fluorescent PAMAM dendrimer as innovative tool for biomedical applications**

*Alessandro Paolini*, Luca Leoni, Ilaria Giannicchi, Zeinab Abbaszadeh, Valentina D’Oria, Francesco Mura, Antonella Dalla Cort, Andrea Masotti*.*

Table S1. The main physical characteristics of the PLA scaffold.

| Scaffold characteristics | Dimensions |
| --- | --- |
| Filament | 0.5 x 0.5 mm |
| Filaments space | 0.6 mm |
| Scaffold | 4.9 x 4.9 x 0.5 mm |
| Scaffold surface | ~ 42.2 mm^2^ |
| Scaffold weight | 6 ± 0.4 mg |

Figure S1. DSC analysis of commercial PLA and 3D-printed scaffold. The glass transition, cold crystallization and fusion temperatures of the two materials have been reported. Data strongly support the similarities of PLA before and after 3D-printing.


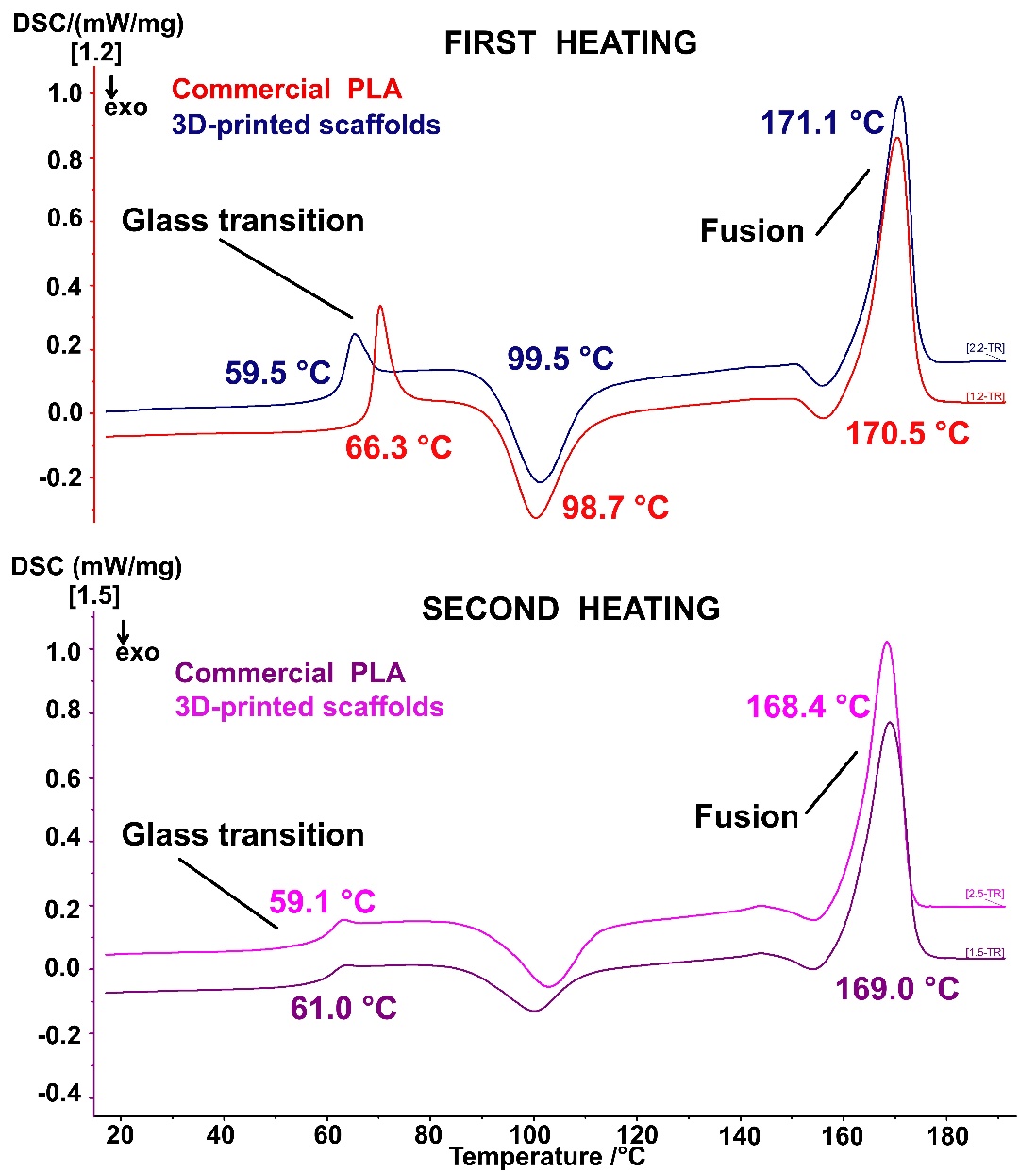


Figure S2. EDX analysis (4 keV, ~300 nm penetration) of 3D-printed PLA scaffolds coated with PAMAM dendrimer (left) and uncoated (right). The white squares indicate the analyzed area (approx. 60x45 µm). Magnification 50.00 Kx.

**Figure S3.** Control HeLa cells (A) treated with FAM-mir-503 (B).

**Figure S4**. Standard solutions at precise concentration of Rho_1%_-PAM in PBS were measured at 580 nm and the plot of the mean of five replicates was reported to obtain the calibration curve. After scaffold incubation, the remaining solution of Rho_1%_-PAM was measured at 580 nm and the value obtained was plotted with calibration curve to obtain the amount of unbound Rho_1%_-PAM. By comparing the initial concentration of PAM-Rho 1% (60 µg) was the amount of PAM-Rho 1% bonded on the scaffold.

| PAM-Rho 1%  Concentrations | Mean 580 nm  values | Standard  deviation |
| --- | --- | --- |
| 0 | 0 | 0 |
| 15 | 0.008 | 0.001 |
| 30 | 0.016 | 0.002 |
| 60 | 0.032 | 0.001 |
| Samples | 0.017 | 0.003 |


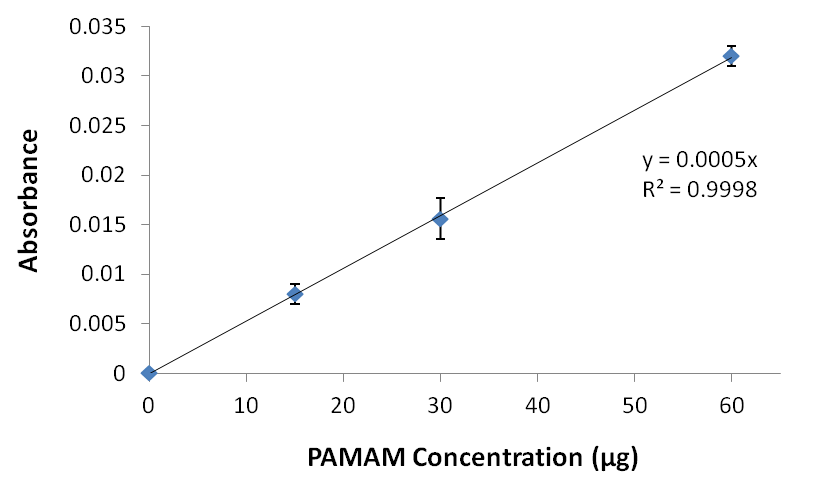

Supplement: Supplementary file 1 — Supporting Information [file 41598_2018_32258_MOESM1_ESM.docx]
